# Famous Poisoners in Fiction

**Published:** 1893-09-30

**Authors:** 


### Famous Poisoners in Fiction.

### VI.—A MOTHER'S LOVE.

"Revenge, tho' sweet, back on itself recoils."—Milton.

This quotation from the works of our English Dante, contains a great truth, and tells of many a life's drama, and of many a bitter hour of agonizing remorse. It is this same truth, so graphically told in this one line by Milton, which is the keynote of the clever history of "The Count of Monte Christo,"* the most famous of all the works of the great French novelist, and the one by far the best known in England. A man most cruelly and unjustly treated by his fellow men is Edmond Dantes, and it is little wonder that when, after fourteen years' imprisonment, he suddenly obtains freedom and fabulous wealth, he determines to act, as he says in one passage of the book, the part of Providence, and to reward his friends and revenge himself on his enemies. Patiently, calmly, silently he works and succeeds, but at the last moment appalled by the terribleness of the consequences of his own acts, he stays his hand and spares the guiltiest of all, but not till he had bitterly suffered and wisely repented. Of Danglar's two accomplices one was dead and the other mad. Here should be noted first one little fact; whilst Edmond Dantes lay pining in prison those men who had complicated him by a wicked plot had all three succeeded in life's struggle; by false means or true they had climbed the ladder of fame, of advancement, of success, and thus their fall was the greater and his revenge more complete.

But even Providence needs a weapon with which to strike, and Dantes had to look about for a suitable tool; this, in M. de Villefort's case, he finds in the beautiful young wife of the *procureur du roi*.

Great indeed is the influence of a word, nay more, of a thought. Many might be inclined to ascribe to hypnotic influence the ease with which the Count de Monte Christo succeeded in gaining his desire, and causing this woman to simply work his will. But the ground was ready for the sin sown, and her heart prepared for the tempter's whisper, for hatred, bitterness, and jealousy had wrought therein those thoughts of ill, which the fatal gift of Monte Christo gave her the power to transform into deeds of evil.

The chapter on toxicology is very fascinating reading to those who care to penetrate into the mystic realm of poison lore. The experiments spoken of as made by the Abbé Adelmonte are interesting, quite apart from the novel. The first, a description of the effect of watering a cabbage with a distillation of arsenic ought to be a warning against eating (anyway without peeling) American apples, if it be true that in the New World on the other side of the Atlantic they do indeed water their apple trees with a dilution of this poison for the sake of destroying the fatal little greenfly and caterpillar. It is quite true that, as studious Mdme. Villefort explains, "arsenic is indelible, indestructible; in what way soever it is absorbed it will be found again in the body of the creature from the moment when it has been taken in sufficient quantity to cause death." Therefore, is it that strychnos colubrina or strychnine is the wisest poison, if not the favourite one, for the would-be poisoner to water the cabbage with. The rabbit ate of this strychnine watered cabbage and died, the fowl ate of the intestines of the rabbit and died also, and yet no symptom of poisoning was apparent on a post mortem being held over the dead bird's body, and not even twelve wise jury fowls could have brought in any other verdict than death by apoplexy.

What the elixir was which the Count of Monte Christo gave to Mdme. de Villefort is not stated, but it must, judging from the accounts given of the murders, have been a preparation of brucine, a most dangerous and violent poison. The system can, as in the case of chloral, arsenic, and opium become so imbued by very small doses of brucine gradually increased, that a sufficient quantity to kill two ordinary men would have little or no effect on a patient habituated to the drug. This fact plays an important part in the novel, not only saving Valentine's life, but defeating the cruel hand which had planned the destruction of weak helpless M. Nortier. It is evident that Dumas has carefully read and studied the properties of this powerful drug, of this medicine yet poison, which may so easily become, as man willeth, either a means of death or of life, a hell-born curse or a heaven-given blessing. The great novelist describes carefully in the chapter headed "The Lemonade," and which contains the horrible account of the death of Barrois, the faithful and beloved old servitor, the effect of brucine on a phial containing a preparation of syrup of violets. "The doctor," we read, "then slowly poured some drops of the lemonade from the decanter into the cup, and in an instant a kind of light cloudy sediment began to form at the bottom of the cup; this sediment first took a blue shade, then from the colour of sapphire it passed to that of opal, and from opal to emerald. Arrived at this last hue, it changed no more." Nearly every poison has some technical means of being thus proved by medical examination, and syrup of violets is one of the tests oftenest used by French doctors.

Two great influences urged on Madame de Villefort to the dread act of murder—even love and jealousy. Love maternal, love—holiest of all emotions—was in her case perverted into a curse; she loved too devotedly, she was greedy, selfish for her boy. He must possess everything, he must have wealth, honour, all that makes life bright and happy. She therefore determines, whether it be the will of God or not, to grasp at these things for her child. The other great motive power which urged her on to crime was jealousy, that vice which is as cruel as the "grave" and implacable as death itself. The beauty, the sweetness, the charms of Valentine but increased her stepmother's jealousy. She hated the young heiress who possessed the wealth she desired for her son. Valentine, she determined, must die, for her death would advantage her darling boy, and not only Valentine, but Madame de S. Meran, and all who stood in the way of her project.

Madame de Villefort is an ideal poisoner. Cold, heartless, determined, she calmly sees those she ruthlessly condemns to death suffer and die without apparently feeling any pangs of conscience until the fear of discovery frightens her, and again, when that has passed

* "The Count of Monte Christo," by Alexander Dumas (*père*).

away she is herself once more, relentless, impassive, self-willed, and determined. One of the cleverest touches in the book is where the woman who has, as she believes, committed four murders plays gaily at ball with her innocent child.

Serpent-like is this beautiful woman with the ice-bound heart, who, in awful grandeur stands out for her devilish cruelty above all other poisoners in the realms of romance, a veritable Marquise de Brinvilliers of the world of fiction, For one so cruelly wicked, not the very tenderest heart can find pity, even for her awful if just doom. Yet, perhaps, with another husband her life might have been different, or if the words of Monte Christo had not been whispered in her ear to act as the lighted match, causing to flare up in a terrible explosion the dynamite of hatred therein hidden, and which might otherwise have remained buried for all time in its dark recesses. Has not every one thought thoughts at times worthy to compete with those which haunted the cruel calculating brain of this beautiful but dangerous woman ?

